# Employees and the National Insurance Act

**Published:** 1912-07-20

**Authors:** 


### NATIONAL INSURANCE ACT DEPARTMENT

### EMPLOYEES AND THE NATIONAL INSURANCE ACT.

### XX.—How Rates of Remuneration Affect Contributions.

In order to relieve the worker who is paid a low rate of wage when such worker has, or is likely to have, persons dependent upon him, the framers of the Act conceived the laudable idea of modifying the rate of contribution payable by the worker. The actual provisions in this matter are set forth in the Second Schedule to the Act, and occupy nearly three times as much space as the statement of the normal contributions and their distribution between the employer and the employee. There was no desire to alter the normal contributions when the worker was a young person without dependents, and consequently the special provisions only apply when the remuneration falls below the sums stated and the worker is twenty-one years of age and upwards. The idea is a good one theoretically, but in practice it is liable to produce a large amount of trouble and inconvenience, and will in many cases, it is to be feared, tend to create strained relations between master and servant.

#### What Remuneration Includes.

The first difficulty is in regard to the amount of the remuneration. The remuneration for services rendered not only includes the pecuniary sums that are paid, but also the value set on board or partial board that may be provided by the employer, and any perquisites that may appertain to the situation. The value to be set on these extras will almost certainly lead to disputes. The general rule of law for valuing such provision is not the amount it may actually cost the employer to provide, but the amount it would cost the worker himself if he had to provide them. In cases of dispute the Insurance Commissioners will decide between the parties.

There are cases in which the remuneration will be less than the money wages, where, for instance, the worker incurs the cost of material or other expenses recognised by the practice of the trade. These will be deducted from the money wages in reckoning the rate of remuneration.

#### How the Rate is to be Calculated.

After the amount of the remuneration has been decided there is the further question of the calculation of the rate. This is necessary because the modification of the rates of contribution is made to depend upon the rate of remuneration per working day. The Act does not prescribe the rules to be followed in making these calculations, but the Insurance Commissioners have published in an official leaflet (Number 18) certain indications of the manner in which the rates are to be calculated. When the employed person is paid by the day the only matter that has to be considered is the value to be set on remuneration other than that paid in money. When, however, the employed person is paid by the week the total earnings received in the week must be divided by the number of days in the week on which work is ordinarily done, in order to obtain the rate of remuneration per day. Apparently, from the example given of a man who ordinarily works five and a half days a week, the number of working days is to be reckoned as six whole days.

#### Wages by Week, by Hour, and by Piece.

When wages are paid less frequently than weekly the rate per week is first to be calculated and the process already described for dealing with the calculation of the rate per day when the wage is payable weekly will be adopted. When the employed person is paid by the hour the rate of remuneration per working day is to be found by multiplying the rate per hour by the number of hours in a working day. In some cases the number of hours in a working day is a definitely recognised figure, and in such circumstances no difficulty can occur, but when there is no such recognised number of hours per day this has first to be ascertained by dividing the number of hours in the week in which work is ordinarily done by the number of working days on which work is ordinarily done.

The calculation that has given most ground for doubt is that connected with ascertaining the rate of remuneration when the worker is paid *by the piece*. The method suggested by the Commissioners is distinctly ingenious, and consists in first finding the actual amount of money per hour which the employed person is receiving in respect of the work for which payment is being made. This has then to be multiplied by the number of hours in an ordinary working day in order to obtain the rate per day.

All these varieties of methods of payment for services rendered will require special calculations week by week, and the preparation of the accounts before the payment of wages will be much more complex than formerly. It would seem that in the case of pieceworkers the sum which the employer is entitled to deduct from wages will vary from week to week.

#### Disputes as to the Rate of Remuneration.

In cases where the employer and the employee are unable to agree as to the amount to be deducted from the employed person's wages in any week, the employer, since the payment of the contributions cannot be postponed, must stamp the card and may take either of two courses: (*a*) he may make the deductions at the lower rate and himself take the necessary steps to obtain the Commissioners' decision, or (*b*) he may make the deductions at the rate which he himself considers to be correct and allow the employed person to take the initiative in the matter of submitting the point to the Commissioners. In this latter case, if the decision is adverse to the employer he will have to make good to the worker any sums which he may have deducted in excess of the correct figure.

INSURANCE OF PRIVATE NURSES.

An important point has just been settled in regard to the insurance of nurses who have their own private practice. The decision is contained in a letter addressed to a maternity nurse who had asked whether it was compulsory for her to be insured, she having her own private practice, and is in the following terms:—

"The National Health Insurance Commissioners are advised that, generally speaking, nurses, working on their own account, will be required to be insured, and the persons by whom they are engaged will, as the employers, be liable for the payment of the contributions. The contributions due from the nurse will be recoverable by deduction from her remuneration. During unemployment it will be open for the nurse to pay the whole contribution herself to avoid reduction of benefits owing to arrears. The only case, generally, in which a nurse will not have to be insured is the case where she receives patients into her own home for treatment."

SANATORIUM BENEFIT.

The only benefit under the Act that can come into immediate operation is sanatorium benefit. All other benefits are deferred for certain waiting periods. It is therefore important that we should inform our readers of the arrangements that have been made in connection with the administration of this particular benefit, and we are dealing with this next week.

### Answers to Correspondents.

PRIVATE NURSES AND INSURANCE.

NURSE L. W., Essex.—It is not clear from your letter that you come within the compulsory provisions of the National Insurance Act. To become an employed contributor you must be employed within the meaning of the Act, and this requires, among other things, that there shall be a contract of service. This contract need not be in writing: it may be verbal only, and either express or implied. If you are bound to render services to the patient you are nursing, and such services are under her power of control, it would seem that your patient is your employer, and that you must therefore be insured provided your remuneration is not at a rate exceeding £160 per annum.

It is possible, however, that the Insurance Commissioners might consider that as you work on your own account you would not have to be insured. If this be so decided you could, nevertheless, become a voluntary contributor, provided your income from all sources does not exceed £160 per annum. If you are unable to decide whether there is a contract of service or not you should apply to a post office for a form on which to furnish the Insurance Commissioners with full particulars, or write direct to the National Health Insurance Commissioners, Buckingham Gate, London, S.W. You should, however, read very carefully the letter to which we refer in our current article. If you are an employed contributor your employer will have to pay 6d. each week for a National Health Insurance stamp and affix this stamp to your contribution card. She will be entitled to deduct 3d. each week from your remuneration. If you become a voluntary contributor you will have to pay the full 6d. a week yourself, or a higher sum if your age is over forty-five. You can obtain a contribution card from any post office or from the Secretary of the Nurses' Insurance Society, 15 Buckingham Street, Strand, London, W.C. We advise you to join this society if you become insured either as a voluntary or an employed contributor.

I. C. M., Camden Road.—See answer to Nurse L. W. above. Your case is almost identical. We are glad to learn that you find THE HOSPITAL useful in securing work from time to time.

S. A. C., Norfolk.—See answer to Nurse L. W. above, and the letter addressed to a maternity nurse by the Insurance Commissioners, to which we refer in our article.

EMPLOYMENT WITHOUT WAGES.

E. M. H., Brighton.—It would seem that both you and your sister are employed persons within the meaning of the Act, even though you do not receive any pecuniary remuneration for your services. You will therefore have to be insured, but your employer will, in the circumstances, have to pay the whole of the contribution without the right of recovering the employee's contribution from yourselves. This case is governed by the Third Schedule to the Act, Clause (8). You can only obtain the full benefits of insurance by joining an approved society. Your sister might be able, as a trained nurse, to gain admittance to the Nurses' Insurance Society, but the disablement from which she suffers might preclude her from becoming a member. As you are yourself, presumably, not a trained nurse you would not be entitled to become a member of this particular society, but your choice of a society should be on the lines indicated in our issue of June 22 (page 298).

K. M. L.—We are much obliged for your letter. You will see that the subject is referred to in our current Insurance Article.

THE NURSES' INSURANCE SOCIETY.

A MATRON of a Cottage Hospital (M.D.) asks us to forward her particulars of the Nurses' Insurance Society. We are unable to comply directly with this request, but we advise her and all other trained nurses who wish for similar information to apply to the Secretary of the Nurses' Insurance Society, 15 Buckingham Street, Strand, W.C.

NOTE.—Questions and Correspondence on all doubtful points are invited, and should be addressed to the Editor, THE HOSPITAL, 28 Southampton Street, Strand, W.C., and marked "Insurance."

